# Detection of Brownian Torque in a Magnetically-Driven Rotating Microsystem

**DOI:** 10.1038/srep21212

**Published:** 2016-02-15

**Authors:** Maria N. Romodina, Evgeny V. Lyubin, Andrey A. Fedyanin

**Affiliations:** 1 Faculty of Physics, Lomonosov Moscow State University, Moscow 119991, Russia

## Abstract

Thermal fluctuations significantly affect the behavior of microscale systems rotating in shear flow, such as microvortexes, microbubbles, rotating micromotors, microactuators and other elements of lab-on-a-chip devices. The influence of Brownian torque on the motion of individual magnetic microparticles in a rotating magnetic field is experimentally determined using optical tweezers. Rotational Brownian motion induces the flattening of the breakdown transition between the synchronous and asynchronous modes of microparticle rotation. The experimental findings regarding microparticle rotation in the presence of Brownian torque are compared with the results of numerical Brownian dynamics simulations.

Brownian motion is the random motion of microparticles suspended in a fluid. The best known and most extensively studied type of Brownian motion is translational movement, which plays a significant role in the behavior of colloids and macromolecules. By contrast, Brownian rotation is the hidden aspect of Brownian motion because it is much more difficult to measure Brownian torques than Brownian forces. There are only a few papers devoted to the detection of the rotational Brownian motion of microobjects, such as anisotropic microparticles^1^,^2^,^3^, optically trapped birefringent quartz microparticles^4^ and macromolecules^5^. Stochastic torques also influence the rotation of microscale systems in liquids, such as micromotors and microvortexes.

Magnetic particles actuated by rotating magnetic fields are commonly used as controllable nano- and micromotors^6^,^7^ and microfluidic mixers^8^. Rotating microparticles are also utilized to twist biological molecules to probe their torsional stiffness^9^ as well as to rotate the surrounding fluid to probe the mechanical properties of liquid media^10^,^11^,^12^. The influence of rotational Brownian motion on the behavior of magnetic microparticles under the action of a rotating magnetic field remains a challenging problem in soft matter physics.

There are two modes of microparticle rotation controlled by the interplay between magnetic and viscous torques^13^,^14^. In magnetic fields rotating at low frequencies, the microparticles rotate synchronously with a rotating magnetic field. Once the frequency of the magnetic field rotation exceeds some critical value *ω*_*crit*_, the microparticle motion becomes asynchronous (see Fig. 1).

The value of the critical frequency depends on the viscosity of the liquid^15^, the strength of the magnetic field and the shape of the microparticles^14^. The responsivity of the critical rotational frequency to changes in liquid properties allows a microparticle to be a tiny physiochemical microsensor of the local properties of a liquid with a volume of a few picoliters^16^,^17^. One fascinating application of such a system is as a biosensor for monitoring the growth and drug susceptibility of an individual bacterium^18^. This approach can be improved by using optical tweezers combined with external magnetic field^19^,^20^ to control the microsensor’s position in space because, in this case, it is possible to perform measurements at different points in the system and to thus obtain the spatial distributions of the studied parameters.

Magnetic microparticle rotation actuated by an external field at rotational frequencies notably higher or lower than the critical value has been intensively studied, both experimentally and theoretically^13^,^14^,^15^,^21^. The influence of an occasional perturbation on the motion of a birefringent cylinder optically rotating with a frequency slightly lower than the critical value has also been determined^22^. This system was found to display a binary (“all-or-none”) response to the input perturbations. However, the question of how Brownian torques affect the rotation of a microparticle is still open, and the behavior of a rotating microparticle near the critical frequency remains unclear. For a system with two stable modes of motion, it is expected that stochastic perturbations would affect the transition between these modes. This effect should be governed by the Langevin parameter, equals to ratio between the energy of the interaction of the microparticle with the magnetic field and the thermal energy^23^.

In this paper, the determination of the influence of Brownian torque on the rotation of a spherical magnetic microparticle using optical tweezers is reported. The experimental dependences of the microparticle rotation on the external magnetic field strength and the rotational frequency clarify the microparticle dynamics near the transition between the synchronous and asynchronous rotational modes. The results are in agreement with numerical Brownian dynamics simulations of microparticle rotation in the presence of Brownian torques.

## Modelling

**Figure 1:**
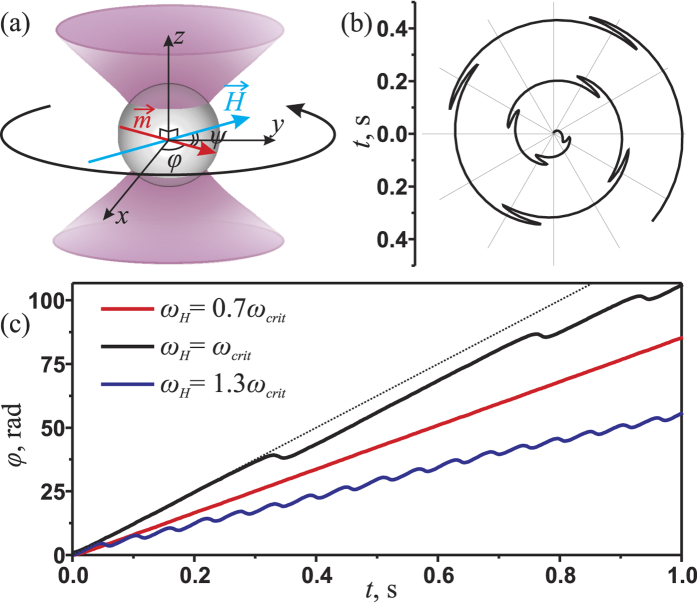
Schematic of the detection of Brownian torque on an optically trapped magnetic microparticle (panel (**a**)). Brownian dynamics simulations of the microparticle’s rotation angle *φ* as a function of time in polar coordinates (panel (**b**)) for *ω*_*H*_ = 1.3*ω*_*crit*_ and in linear coordinates (panel (**c**)) for *ω*_*H*_ = *ω*_*crit*_ (black curve), *ω*_*H*_ = 0.7*ω*_*crit*_ (red curve), and *ω*_*H*_ = 1.3*ω*_*crit*_ (blue curve); the dashed line represents *φ* = *ω*_*crit*_*t*. Langevin parameter equals to 250.

The equation for the angular motion of a spherical magnetic microparticle in a liquid in the presence of an external rotating magnetic field can be written in the following form^16^,^24^:


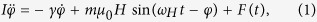


where *φ* is the angle corresponding to the turning of the particle in the plane of magnetic field rotation, *I* is the moment of inertia of the microparticle, *γ* = 8*πηa*^3^ is the coefficient of viscous torque for a spherical particle of radius *a* in a liquid with viscosity *η*, *m* is the magnetic moment of the particle, *H* is the magnetic field strength, *ω*_*H*_ is the angular frequency of magnetic field rotation, *t* is time and *F*(*t*) is the Brownian torque (see Fig. 1a). Langevin parameter for this system can be written as following: *ξ* = *mμ*_0_*H*/*k*_*B*_*T*, where *T* is the temperature, *k*_*B*_ is the Boltzmann constant. In most papers^13^,^14^,^16^, inertia and Brownian torques are assumed to be negligible in microparticle rotation. In this approximation, the microparticle rotation is controlled by the interplay between magnetic and viscous torques and exhibits two distinct modes of motion: synchronous and asynchronous rotation. Synchronous rotation occurs for low *ω*_*H*_ values because the magnetic and viscous torques are in equilibrium and the phase difference between the particle and the magnetic field *ψ* is constant and equals to arcsin(*ω*_*H*_/*ω*_*crit*_), where the critical frequency of the transition from the synchronous to the asynchronous rotational mode, *ω*_*crit*_, is written as follows^16^:


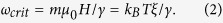


The microparticle’s angle *φ* scales linearly in time, *φ*(*t*) = *ω*_*H*_*t* − *ψ*, and the phase difference *ψ* increases with an increase in the frequency of magnetic field rotation, *ω*_*H*_. When *ω*_*H*_ approaches *ω*_*crit*_, the phase delay becomes equal to *π*/2 and the particle rotation becomes asynchronous: the microparticle undergoes a periodic sequence of forward and backward turns, rotating, on average, in the same direction as the field (see Fig. 1b) but with a frequency 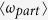 lower than *ω*_*H*_. When 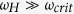, the mean angular frequency of microparticle rotation 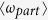 is given by the following equation^16^:


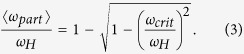


The influence of rotational Brownian motion on the microparticle rotation is numerically revealed by Brownian dynamics simulations^25^. Brownian torque is assumed to be a Gaussian white-noise phenomenon with the first moment 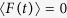 and second moment 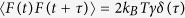, *τ* is the time delay, and *δ* is the Dirac function^26^. The inertia term is neglected in Eq. (1) because the microparticle is rotating in a liquid with a small Reynolds number^16^,^26^. Using Kramers-Moyal coefficients defined for the presence of a stochastic torque, Eq. (1) can be reduced to the following iterations^27^:


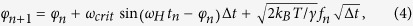


where *f*_*n*_ is a random Gaussian function with a mean value of zero and a dispersion equals to 1. The time step between iterations in simulations process, Δ*t*, was equal to 10^−6^ s, and the number of iterations was equal to 10^9^.

The numerical results for microparticle rotation are shown in Fig. 1(c) for various values of *ω*_*H*_. The particle’s turn angle *φ* is linearly dependent on time when *ω*_*H*_ = 0.7*ω*_*crit*_. When *ω*_*H*_ = 1.3*ω*_*crit*_, the angle *φ* periodically oscillates up and down, but on average, the value of the angle is increasing. For *ω*_*H*_ = *ω*_*crit*_, the *φ*(*t*) dependence is still linear; however, occasional non-periodic backward turns arise in the particle’s motion. These spontaneous backward turns are a result of the action of the rotational Brownian torque, whose is now sufficient for system switching between the synchronous and asynchronous rotational modes. The rotational mode of the microparticle stochastically switches from synchronous to asynchronous and vice versa. The corresponding forward and backward turns of the particle can be clearly observed in polar coordinates, as shown in Fig. 1(b). The average turnaround time of the particle, *t*_*turn*_, can be derived from the *φ*(*t*) dependence calculated for each value of the external magnetic field’s rotational frequency *ω*_*H*_. The normalized mean angular frequency of the particle rotation, 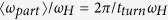, can then be compared with the experimental data.

## Methods

**Figure 2:**
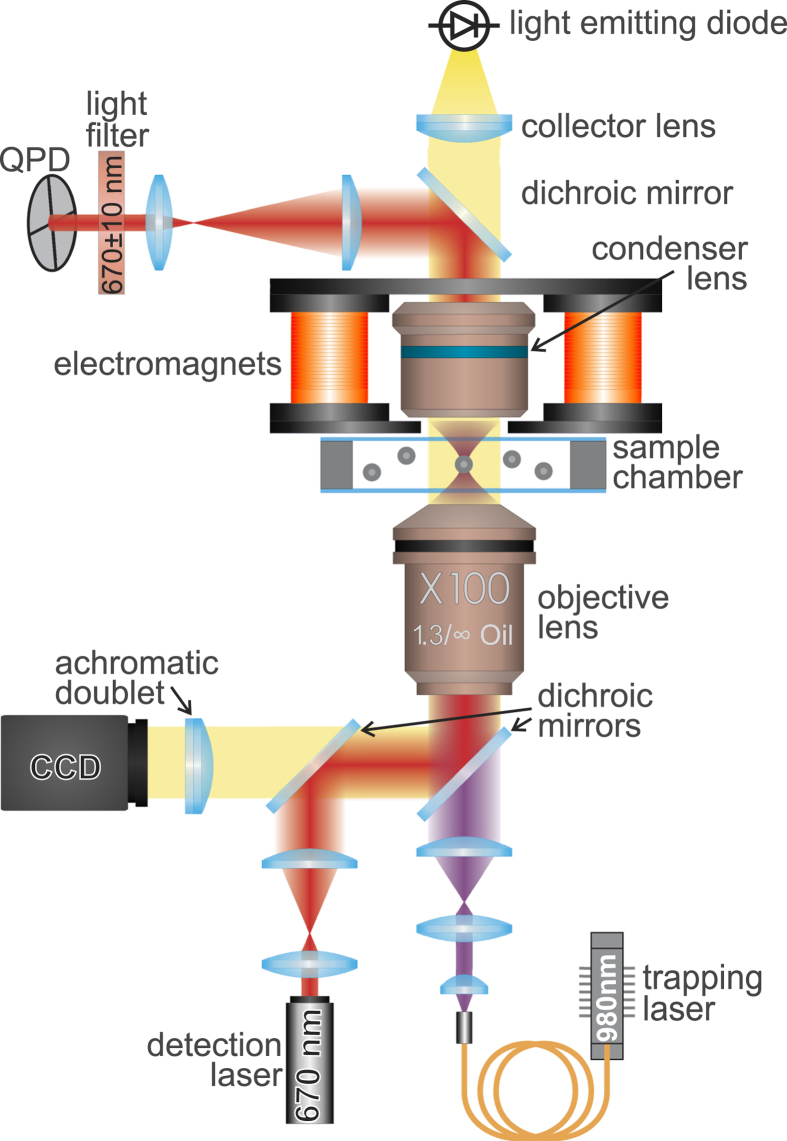
Experimental setup to use optical tweezers with electromagnets for the study of microparticle rotation in an external magnetic field.

**Figure 3:**
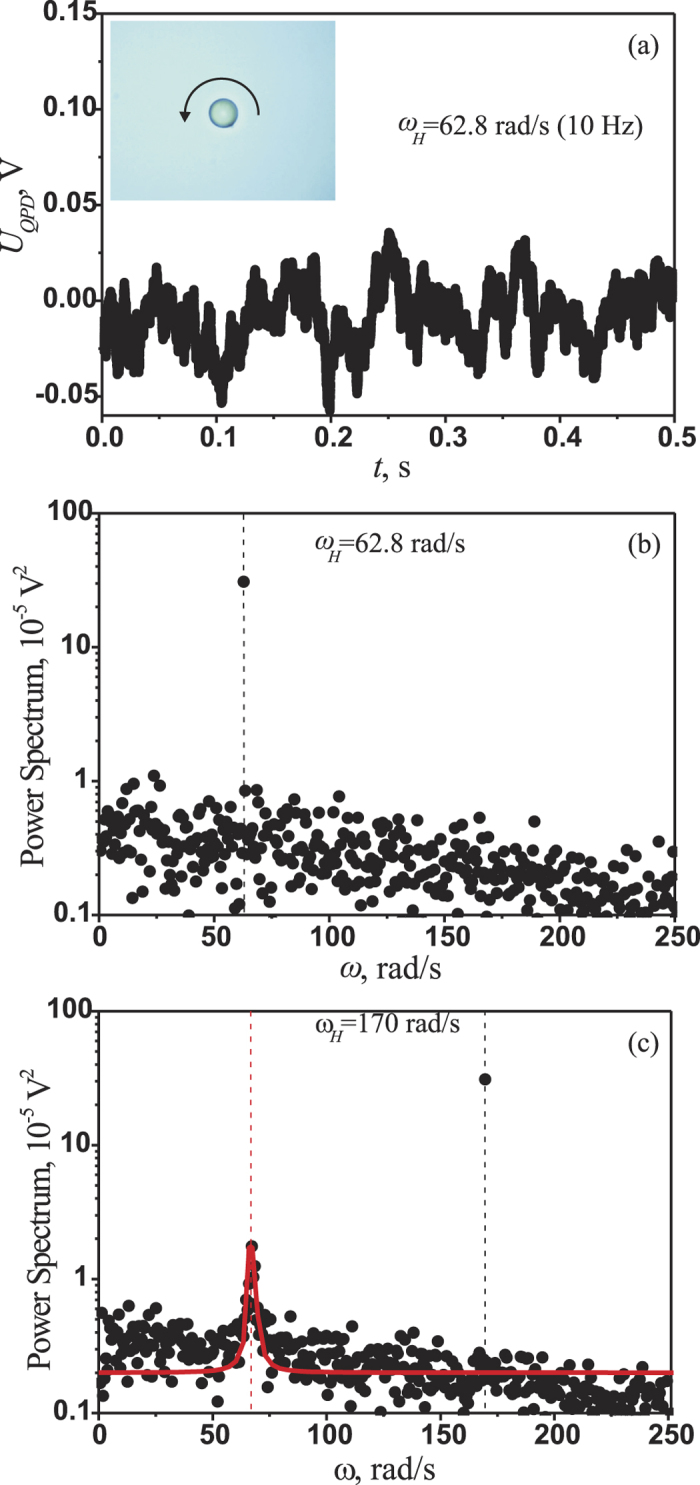
Experimental results for a single optically trapped magnetic microparticle in an external rotating magnetic field. The critical frequency of the microparticle rotation was approximately 136.3 rad/s, the magnetic field strength was equal to 21 Oe, and the trapping laser power was 10 mW. Inset: A microimage of a trapped microparticle. The panels show the time dependence of the QPD voltage for a magnetic field rotation frequency of 62.8 rad/s, or 10 Hz (panel (**a**)); the power spectrum of the QPD voltage for a magnetic field rotation frequency of 62.8 rad/s (panel (**b**)); and the power spectrum of the QPD voltage for a magnetic field rotation frequency of 170 rad/s (panel (**c**)). The red curve represents fit performed using the Lorenz function.

Single magnetic microparticle rotation was studied using optical tweezers combined with four electromagnets (see Fig. 2). The studied microparticles were 3 *μ*m in diameter with standard deviation <5% and made from polystyrene with magnetite nanograins (PMPEG-3.0COOH, Kisker Biotech. GmbH, Germany). Approximately 40 *μ*l of a water suspension of the particles with a concentration of 50 *μ*g/ml was placed into a hermetic chamber at room temperature (24 °C). The chamber was formed using two 0.1-mm-thick cover slips separated by a 0.15-mm gap and placed into the operating area of the optical tweezers. The microparticle suspension contained a small amount of albumin (0.1%) to prevent the microparticles from adhering to the surfaces of the sample chamber. The optically trapped microparticle was localized at a distance of 20 *μ*m above the bottom cover slip. The trap was formed by focusing a diode laser beam with a wavelength of 980 nm using a high-numerical-aperture objective (NA = 1.3). The fluctuations of trapping beam power were smaller than 0.5%. For the visualization of the trapping area, a white-light-emitting diode was used to illuminate the sample chamber through a condenser lens, and the signal was collected using a charge-coupled device (CCD) camera. A typical snapshot of a trapped microparticle is shown in Fig. 3(a).

The microparticle was rotated by applying an external magnetic field to the sample using four compact independent electromagnets, each with a tip-pole expansion. The magnetic components were designed to maintain a constant magnetic field flow in the sample area. A sinusoidal current was applied to each electromagnet, with a phase delay between currents equal to *π*/2 for neighboring electromagnets and *π* for opposing electromagnets. The magnetic field vector was rotated in the plane parallel to the cover slips of the sample chamber. The frequency and amplitude of the magnetic field rotation were controlled by a computer; for the technical details, see ref. 28. The maximum magnetic field was approximately 3 kA/m. An additional laser with a wavelength of 670 nm was used to detect the microparticle rotation.

The radiation from the laser was scattered onto the microparticle, collected by the condenser and registered using a quadrant photodiode (QPD). The microparticle had a slightly non-ideal spherical form (see Fig. 3(a)), and the laser spot scattered by the microparticle was also non-symmetrical in shape. Thus, for the rotating microparticle, the voltage difference across the QPD contacts, called the QPD voltage, was modulated by the frequency of the particle rotation; see Fig. 3(a). The extracted power spectrum of the QPD voltage exhibited a clear maximum, with a peak position corresponding to the frequency of the microparticle motion. Figure 3(b,c) represent typical experimental power spectra of the QPD voltage for different values of the rotational frequency of the magnetic field. The power spectrum of the QPD voltage in the case of uniform microparticle rotation, i.e., at frequencies lower than the critical value, is shown in Fig. 3(b). It possesses a typical maximum at the frequency of the microparticle rotation (62.8 rad/s, or 10 Hz). The power spectrum of the QPD voltage measured for the non-uniform rotation mode for a frequency of magnetic field rotation higher than the critical frequency is shown in Fig. 3(c). The power spectrum exhibits two typical bright maxima: one at the frequency of the external field rotation at 170 rad/s and another at the frequency of complete microparticle revolutions at 67 ± 2 rad/s. The uncertainty of complete microparticle rotation frequency is estimated as a half width at half maximum of the Lorenz fitting function. The normalized mean frequency of microparticle rotation is calculated as the ratio of the frequencies of these peaks, 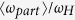. The value and the error of critical frequency *ω*_*crit*_ = 136.3 ± 0.9 rad/s are obtained by fitting Eq. (3) to experimental data.

## Results and Discussion

**Figure 4:**
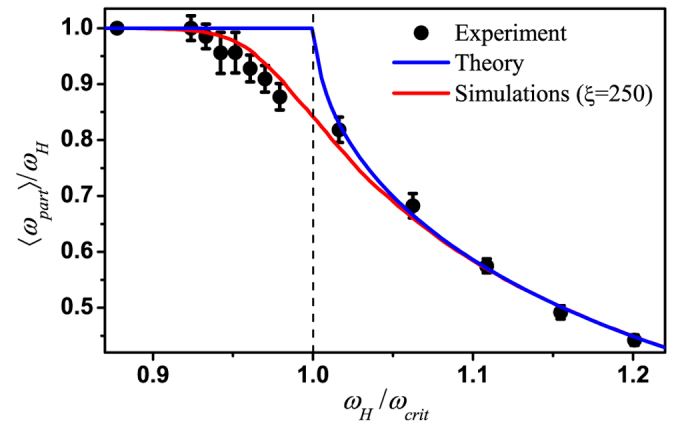
Normalized mean rotational frequency of the microparticles as a function of the rotation rate of the external magnetic field. The experimental data points are represented by circles. The blue curve shows the theoretical dependence given by Eq. (3), and the red curve illustrates the results of the Brownian dynamics simulation for fitting Langevin parameter *ξ* = 250. The critical frequency of microparticle rotation was *ω*_*crit*_ = 136.3 rad/s, the magnetic field strength was *H* = 1070 A/m, and the laser power in the chamber was *P* = 5.7 mW.

The rotation of a single magnetic microparticle was experimentally studied using optical tweezers. The experimentally measured dependence of the normalized mean frequency of the microparticle rotation on the rotational frequency of the magnetic field is shown in Fig. 4. When *ω*_*H*_ is low, the microparticles rotate synchronously with the external magnetic field. For *ω*_*H*_ > *ω*_*crit*_, the average particle rotation rate decreases as the rotational frequency of the magnetic field increases.

The transition from the synchronous to the asynchronous mode of Brownian particle rotation exhibits a smooth shape without any fracture at *ω*_*H*_ = *ω*_*crit*_ because the effect of the Brownian torque on the microparticle rotation leads to fluctuations in the critical frequency. The results of the Brownian dynamics simulations of microparticle rotation are represented by the red curve in Fig. 4, which illustrates the major role played by the Brownian torque in modifying the shape of the transition between the rotational modes.

**Figure 5:**
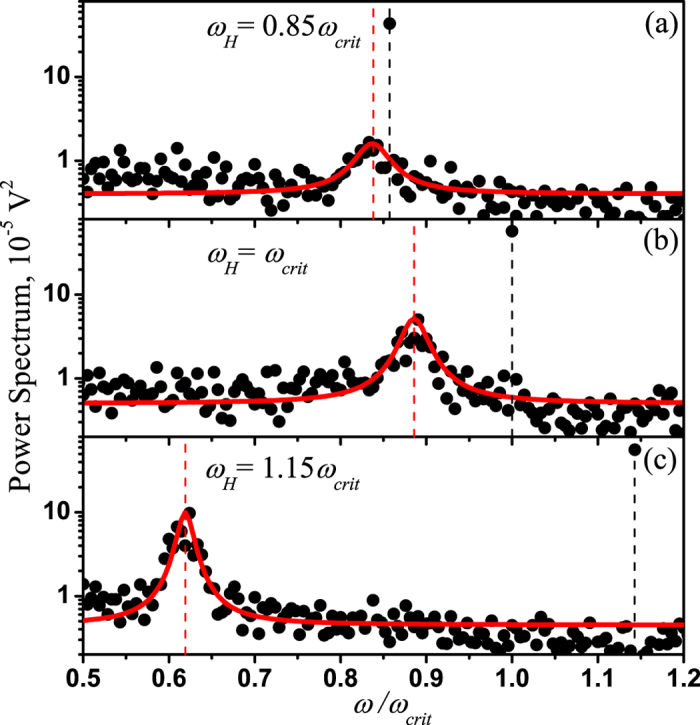
Experimental power spectra of the QPD voltage signals measured with the magnetic field rotating at a frequency near the critical point. The rotational frequencies of the magnetic field were *ω*_*H*_ = 0.85*ω*_*crit*_ (panel (**a**)), *ω*_*H*_ = *ω*_*crit*_ (panel (**b**)), and *ω*_*H*_ = 1.15*ω*_*crit*_ (panel (**c**)). The red curves represent fits performed using the Lorenz function. The critical frequency was *ω*_*crit*_ = 131.9 rad/s.

The stochastic properties of the microparticle rotation near the critical point can be determined from the frequency dependence of the power spectra of the quadrant photodiode (QPD) signals, which are shown in Fig. 5. Asynchronous motion manifests as a peak in the power spectrum of the QPD signal, which becomes higher and narrower with increasing frequency of the magnetic field rotation. When *ω*_*H*_ < *ω*_*crit*_, the particle rotation is not strictly uniform and the peak position shifts toward higher frequencies as *ω*_*H*_ increases (see Fig. 5(a)). For *ω*_*H*_ > *ω*_*crit*_, the peak shifts to the left (see Fig. 5(c)).

**Figure 6:**
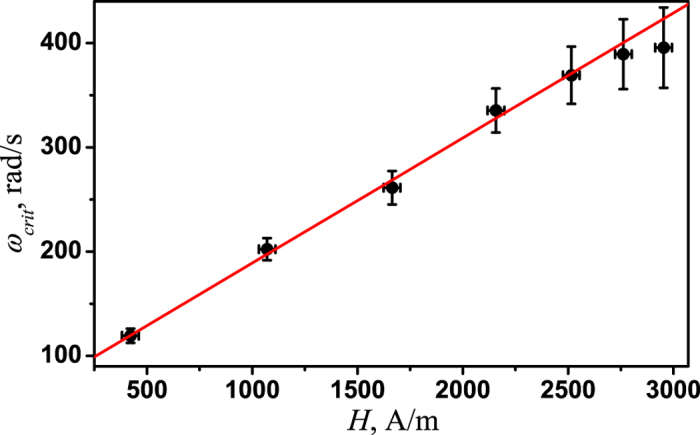
Typical dependence of the critical frequency of microparticle rotation on the magnetic field strength (black circles) and the results of fitting Eq. (2) to the data (red line). The power of the trapping laser was *P* = 5.7 mW.

The transition region shifts toward higher frequencies with an increase in the magnetic field strength. Typical critical frequency is plotted as a function of the magnetic field amplitude in the Fig. 6, showing the linear dependence throughout the entire range of available magnetic field values. Because the critical frequency, according to Eq. (2), is the product of the magnetic field strength and the particle’s magnetic moment, a linear dependence of the critical frequency on the magnetic field strength indicates that the microparticle has a constant part of magnetic moment and that the effect of the paramagnetic part of the magnetic moment on the microparticle rotation is negligible. The constant part of magnetic moment value estimated from a linear fit to the data is (8 ± 0.2) fA · m^2^.

**Figure 7:**
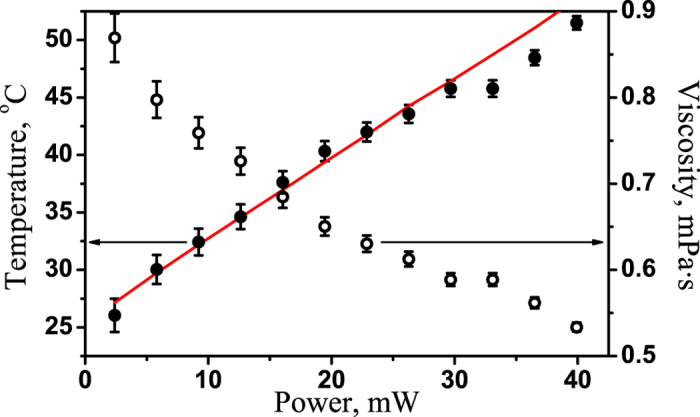
Experimental results regarding the temperature of the microparticle surface (filled circles) and the viscosity of the liquid around the microparticle (open circles) as functions of the trapping laser power, *H* = 1070 A/m. The line represents a linear fit to the data.

Finally, we have shown that measurements of the critical frequency of the transition between rotational modes allow for the extraction of the temperature of a microparticle and the local viscosity of its surrounding medium. The dependence 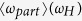 measured for various values of the trapping laser power, *P*, indicates that the critical frequency increases with increasing *P*. The shift in the critical frequency is due to the heating of the trapped microparticles under the laser radiation. The magnetite nanograins embedded in the polystyrene microparticles slightly absorb the trapping light, causing the temperatures of the microparticle and the surrounding medium to increase. Heating the liquid near the particle changes the water viscosity *γ*(*T*), and the critical frequency 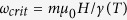 changes also. Thus, the dependence *ω*_*crit*_(*P*) indicates the laser power dependences of the local liquid viscosity of the liquid around the trapped magnetic microparticle, as shown in Fig. 7 using open circles. The effective temperature of the liquid around the trapped magnetic microparticle was estimated using empirical dependence of water viscosity on temperature^29^ and shown in Fig. 7 using filled circles.

These dependences exhibit linear behaviors up to 30 mW. For higher trapping laser powers, the liquid temperature becomes higher than 45 °C, which leads to the folding of the albumin suspended in the water, added to prevent the microparticles from adhering to the surfaces of the sample chamber; this may be the reason for the mismatch between the linear fit and the experimental results for *P* > 30 mW.

## Conclusions

In conclusion, the influence of Brownian torque on optically trapped magnetic microparticles in a rotating magnetic field was studied. The transition between the synchronous and asynchronous modes of particle rotation exhibits a smooth shape without any fracture because of the presence of Brownian torque. The results of numerical Brownian dynamics simulations of microparticle rotation in the presence of a stochastic thermal torque are consistent with the experimental data. The results of the experiment and simulations offer insights into the fundamental Brownian rotation process, which are potentially useful for understanding the diffusion of anisotropic macromolecules and for constructing microscale motors and other devices. The influence of Brownian torque should be taken into account in the analysis of various medical and technical systems. Rotating magnetic microparticles in optical traps can be used as local microscale heaters and mixers for liquids with volumes on the order of a few picoliters. The temperature of such a trapped microparticle can be controlled with a precision of approximately 1 °C, which may be very useful for studying thermal effects in a variety of biological and chemical microsystems.

## Additional Information

**How to cite this article**: Romodina, M. N. *et al.* Detection of Brownian Torque in a Magnetically-Driven Rotating Microsystem. *Sci. Rep.* **6**, 21212; doi: 10.1038/srep21212 (2016).
